# An accurate mathematical model predicting number of dengue cases in tropics

**DOI:** 10.1371/journal.pntd.0009756

**Published:** 2021-11-08

**Authors:** Chathurangi Edussuriya, Sampath Deegalla, Indika Gawarammana

**Affiliations:** 1 Department of Computer Engineering, Faculty of Engineering, University of Peradeniya, Sri Lanka; 2 Department of Medicine, Faculty of Medicine, University of Peradeniya, Sri Lanka; Australian Red Cross Lifelood, AUSTRALIA

## Abstract

Dengue fever is a systemic viral infection of epidemic proportions in tropical countries. The incidence of dengue fever is ever increasing and has doubled over the last few decades. Estimated 50million new cases are detected each year and close to 10000 deaths occur each year. Epidemics are unpredictable and unprecedented. When epidemics occur, health services are over whelmed leading to overcrowding of hospitals. At present there is no evidence that dengue epidemics can be predicted. Since the breeding of the dengue mosquito is directly influenced by environmental factors, it is plausible that epidemics could be predicted using weather data. We hypothesized that there is a mathematical relationship between incidence of dengue fever and environmental factors and if such relationship exists, new cases of dengue fever in the succeeding months can be predicted using weather data of the current month. We developed a mathematical model using machine learning technique. We used Island wide dengue epidemiology data, weather data and population density in developing the model. We used incidence of dengue fever, average rain fall, humidity, wind speed, temperature and population density of each district in the model. We found that the model is able to predict the incidence of dengue fever of a given month in a given district with precision (RMSE between 18- 35.3). Further, using weather data of a given month, the number of cases of dengue in succeeding months too can be predicted with precision (RMSE 10.4—30). Health authorities can use existing weather data in predicting epidemics in the immediate future and therefore measures to prevent new cases can be taken and more importantly the authorities can prepare local authorities for outbreaks.

## Author summary

Dengue fever is a systemic viral infection of epidemic proportions in tropical countries. The incidence of dengue fever is ever increasing and has doubled over the last few decades. Estimated 50 million new cases are detected each year and close to 10000 deaths occur each year. Epidemics are unpredictable and unprecedented. When epidemics occur, health services are over whelmed leading to overcrowding of hospitals. At present there is no evidence that dengue epidemics can be predicted. We developed a mathematical model using machine learning technique to predict dengue epidemics. We used Island wide dengue epidemiology data, weather data and population density in developing the model. We found that the model is able to predict the incidence of dengue fever of a given month in a given district with precision. Further, using weather data of a given month, the number of cases of dengue in succeeding months too can be predicted with precision. Health authorities can use existing weather data in predicting epidemics in the immediate future and therefore measures to prevent new cases can be taken and more importantly the authorities can prepare local authorities for outbreaks.

## Introduction

Dengue is a systemic viral infection transmitted between humans by Aedes mosquitoes with no known cure. It is also the commonest arboviral illness in the world. Dengue fever is endemic in many Asian countries and it has been estimated that 390 million people are infected each year [1]. The incidence of dengue doubled in each decade between 1990 and 2013, from 8.3 million (3.3 million-17.2 million) apparent cases in 1990, to 58.4 million (23.6 million-121.9 million) apparent cases in 2013 [2] Once infected, over an incubation period of 3-10 days the typical symptoms of dengue fever including severe headache, arthralgia, myalgia, rashes and hemorrhagic manifestations begin to develop [3]. The clinical and biochemical profile of dengue fever is well published [4], [5], [6]. Though many of the cases end in uncomplicated Dengue Fever (DF), some may progress to a more severe form, namely Dengue Hemorrhagic Fever (DHF) which usually occurs soon after the end of the febrile phase and results in plasma leakage. If plasma leakage becomes severe, with hypovolemic shock, the disease may progress to Dengue Shock Syndrome (DSS) which is potentially fatal. The mortality is less than 0.4% [7], [8] The exact number of global deaths from dengue fever is unknown, however it was estimated that an average of 9221 dengue deaths occurred per year between the years 1990 and 2013. Dengue outbreaks are unpredictable. When epidemics occur, hospitals are over crowded and resources become stretched to the maximum. At present there are no accepted methods to predict dengue outbreaks in Sri Lanka. If dengue outbreaks can be predicted, measures to prevent such outbreaks can be implemented. Further, health services can be pre-warned and necessary measures can be implemented to absorb unprecedented numbers of dengue patients during such outbreaks. Weather factors (temperature, humidity, rainfall, wind speed) affect the growth of the mosquito density [9]. When conditions are favorable, the mosquito density increases. The increase in rainfall causes accumulation of sewerage and waste management of the country is usually overwhelmed. Dengue mosquitoes lay eggs where water is collected and during rainy seasons breading places become abundant. When the human population density is high the amount of waste disposal also increases and thus more breeding places are created. Existence of the dengue virus in the environment and the mosquito density influence the number of mosquitoes carrying the dengue virus. High temperature is associated with drying of flowing water sources into small pools of stagnant water which is a favorable condition for the dengue mosquitoes to complete life cycle. On the other hand, some larvae may be vulnerable to extremely high environmental temperatures and thus environment temperatures must have both a negative and a positive impact on the mosquito density and number of dengue cases. Machine learning approaches are a new tool which has had a significant impact on bio-informatics in many aspects. Genomics, proteomics, microarrays and disease predictions are some of the instances where machine leaning has been used successfully. However, it has not been successfully used in predicting dengue epidemics so far in Sri Lanka. We conducted a time series analysis using different models for temporal data analysis. We explored a new mechanism to optimize the hyper parameters of a time series model. A given set of factors known to affect dengue epidemiology were used to develop the model.

### Objective

The objective of this research was to study if a mathematical relationship exists between the number of cases of dengue and average rain fall, humidity, wind speed, temperature and population density.

Specific objectives are listed as follows:

1. To develop a model to predict number of cases of a given month of a given district using existing data.
2. To develop a predictive model of number of cases of succeeding months using data of an index month

## Methodology

All the districts in Sri Lanka were selected. We collected epidemiological data of dengue fever from all districts between January 2010 to March 2019. Data was accessed from the public domain of Ministry of health at the official website [10]. District wise, weather data was accessed under license from the department of meteorology of Sri Lanka. The latter included average rain fall, humidity, wind speed and temperature. Data on population density was accessed from report of the central bank of Sri Lanka [11]. The relationship between these conditions were analyzed in a district wise manner. Hence the models were built separately for each district in the analysis.

### Assumption

**Fig 1:**
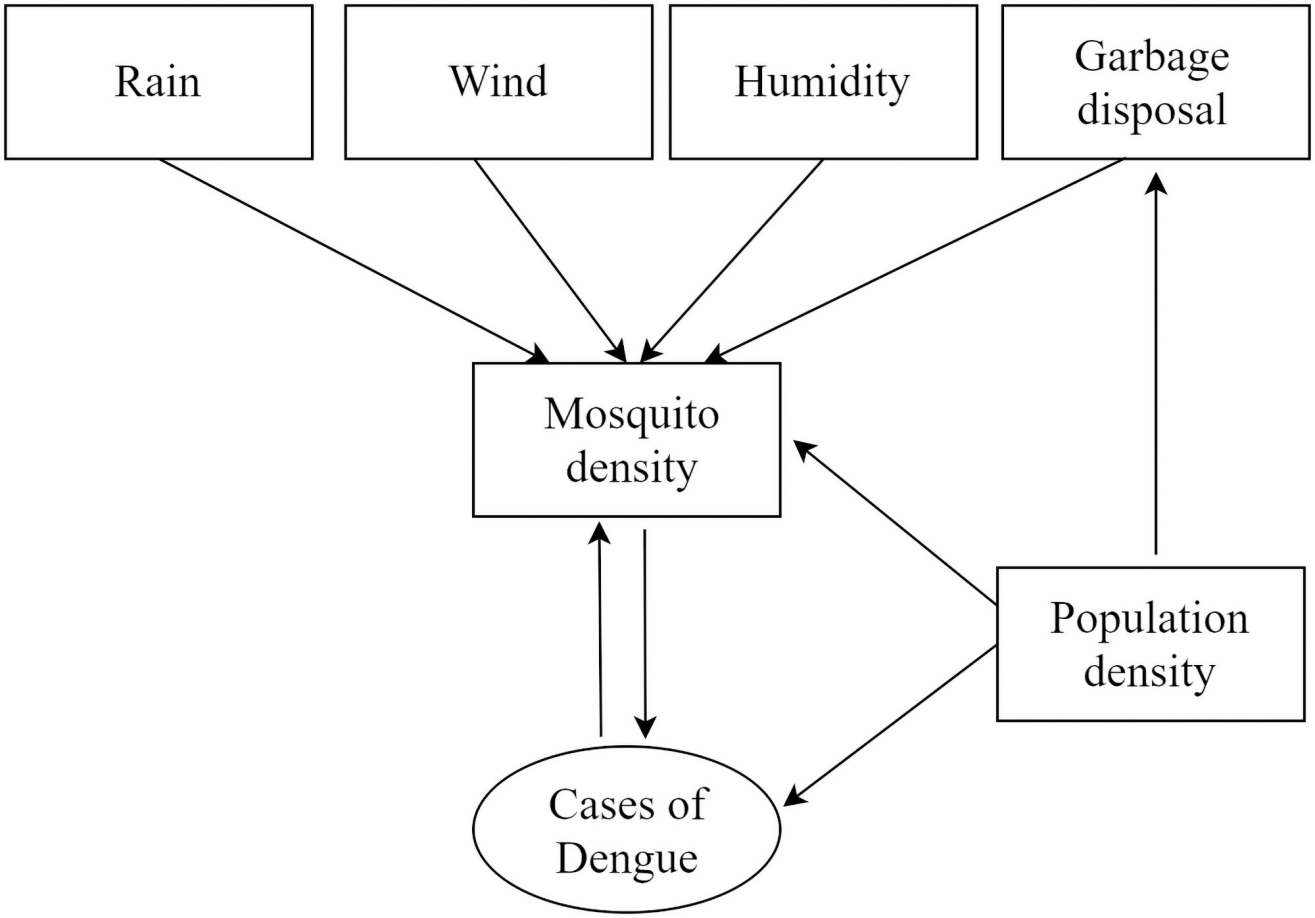
The relationships between the factors effecting the number of dengue cases.

We assumed, based on published evidence, that Rain fall, humidity, wind speed, garbage disposal, sewage and water management, temperature and population density has direct and indirect relationship with the density of mosquitoes and therefore number of cases of dengue. Population density indirectly favors breeding of mosquitoes through a combination of compromised drainage system, cluttered garbage collection and densely packed housing. Hence, in developing the model we assumed that dengue mosquito density and therefore number of cases of dengue had a close relationship as depicted in Fig 1 [9].

### Data collection

As this research is conducted to a comprehensive analysis of dengue outbreaks, all the districts in Sri Lanka were considered. Rainfall, humidity, wind speed, temperature were considered as the weather factors. The weather data were collected from the meteorological department of Sri Lanka. Unfortunately, the meteorology department did not have data on 6 districts and were excluded from the analysis. We analyzed data during the 10-year period from January 2010 to March 2019. Data on the number of dengue victims were collected from the epidemiology unit of ministry of health of Sri Lanka [11]. Evolution of population density data were collected by referring the economics and social statistics of Sri Lanka maintained by the Central Bank of Sri Lanka [10].

### Building the model

For this research, machine learning was used. Machine learning is the mechanism of learning on a given set of data using different models which are built using algorithms to identify patterns and inference instead of following an explicit set of instructions [12]. Machine learning focus more on having the maximum performance of the analysis (accuracy, prediction…) while statistical modeling focus more on finding relationships between different input variables and the importance between the relationships. As the first step of the machine learning approach, the data is pre-processed.

### Pre-processing of data

In the data obtained by the meteorological department, the data of humidity from 2014—2019 were not recorded. Due to the large number of missing data points, humidity factor was removed from the analysis. Then the other missing values were handled by adding the average mean values of each district in a certain year. Then the input features and the dependent variable considered at a particular model were fit into a scale between 0,1 to avoid biasing of the data. The numerical values of population density are around 10000 while the temperature is below 100. If the data is not fit into a scale there would be a high bias of data towards one factor.

### Model

The data are recorded as monthly observations and statistical analysis. This research will be used to predict dengue outbreaks in an upcoming month with a time lag. Hence this analysis can be considered as a time series analysis. For a time series analysis, Support Vector Regression (SVR) [13], Multi-Layer Perceptron (MLP) [14] neural networks can be used. The best possible algorithm which can be used for this kind of time series analysis is the Long Short-Term Memory neural network. [15]

#### Long Short-Term Memory neural network—LSTM

Long short-Term Memory is a type of a recurrent neural network created by SeppHochreiter and Jürgen Schmidhuber in 1997 [16]. A neural network is a type of machine learning model which is inspired by the biological neural network. [17] The neural network contains a nodes or units which mimic the functionality of a neuron. In a recurrent neural network (RNN), the hidden state of the previous step is passed to the next node. [18] In the RNN the gradient value of the loss function reduces exponentially over the time. This is called as the vanishing gradient problem [18]. To overcome shortcomings of RNN, Long Short-Term Memory neural network was introduced. In this network, memory cells are introduced which can hold the data for a long time.

**Fig 2:**
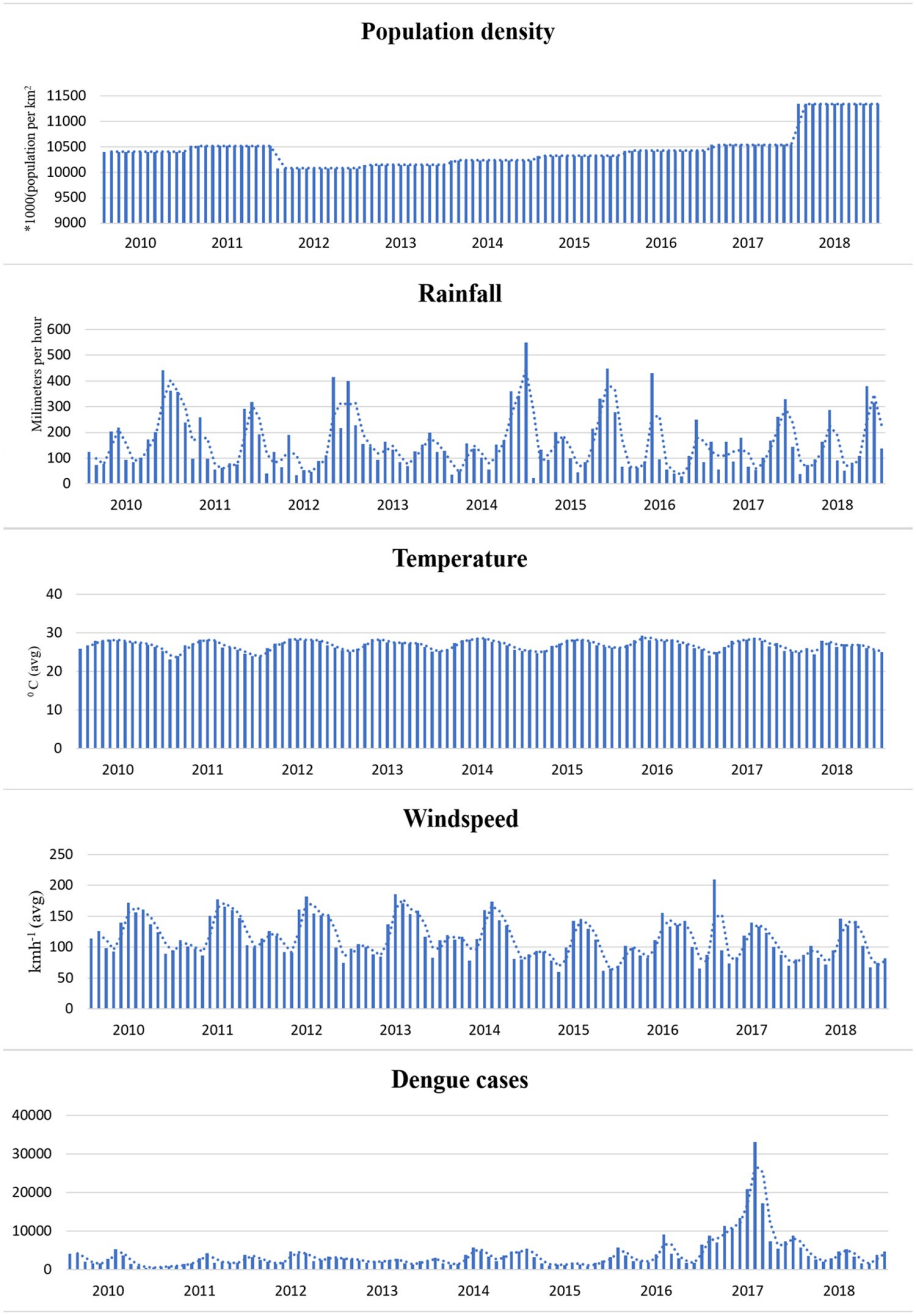
Architecture and the functionality of the LSTM node.

The basic function unit of LSTM unit is given in the Fig 2. In one unit, the result value from the previous block(*h*_*t*−1_) and the result of the previous block is fed in with the input value of the block. The result and the hidden value of the considered block(*h*_*t*_) is fed into the next block(*h*_*t*+1_) As this is studying time series data, the result and the hidden state of the previous block makes a big impact on the result. Sigma and tan functions are considered as the activation functions. An activation function gives an output to a given input with the function included. In a tanh function, the tan value related to the input value is taken as the output. All the input values could be mapped inside the range considered using the activation function. At different junctions, unit multiplication and unit addition are performed on the input data as shown in the diagram.

The equations which are used by one cell for the calculations are shown below [19]. In this research the LSTM model is implemented using the Keras library with tensorflow backend [20].

$$\begin{array}{cc} f_{t} & =\sigma (W_{f}.\left(h_{t-1},x_{t}\right)+W_{f,bias})\\ i_{t} & =\sigma (W_{i}.\left(h_{t-1},x_{t}\right)+W_{fi,bias})\\ C_{t}^{\prime } & =tanh(W_{c}.\left(h_{t-1},x_{t}\right)+W_{c,bias})\\ C_{t} & =f_{t}*C_{t-1}+i_{t}*C_{t}\\ O_{t} & =\sigma (W_{o}.\left(h_{t-1},x_{t}\right)+W_{o,bias}\\ h_{t} & =O_{t}*tanh\left(C_{t}\right) \end{array}$$ (1)

When building the model using LSTM, the hyper parameters of the model have to be chosen. The hyper parameters can simply be said as the settings of a model which would affect the functionality of the model greatly. If we consider 10 hyperparameters and each hyper parameter having 3 values, 3^10^ combinations could be found. LSTM network has a huge computational cost due to the high complexity of the model. If we are going to find the parameters using brute force attack, it will take a huge amount of time for the computation. This is called the Grid Search analysis. Random Search analysis could also be performed. But it cannot guarantee that the optimal solution neither. Hence, the search for optimization algorithms to tune hyper parameters begun. Optimization algorithm is an algorithm which would find the optimal, best solution in a given set of solutions [21]. In optimization algorithm, recently more focus has been given to nature inspired optimization algorithms. In nature inspired algorithms, the mechanisms or phenomenal occurrences in the nature are built into a mathematical algorithm to find the best solution [22]. some nature inspired optimization algorithms are, Genetic Algorithm (GA): inspired by the phenomena of genes, Particle Swarm Optimization (PSO): inspired by flock of birds or school of fish [23]. Grey Wolf Optimization (GWO) is a newly introduced nature inspired optimization algorithm which has proved high performance in optimization [24].

#### Grey Wolf Optimization

Grey Wolf Optimization (GWO) is created by Mirjalili et al [25] in 2014 which mimics the hunting mechanism used by a pack of wolves. In the social hierarchy of wolves, alpha wolf is the leader of the pack. Sleeping place, hunting location are decided. The beta Wolf, the next layer of the social hierarchy helps alpha wolves in decision making. In the next layer, delta wolves are found. The last layer of the social hierarchy is the omega wolves. Omega wolves are allowed to eat only after the other wolves have fed. In the mathematical model of the GWO algorithm, this social hierarchy as well as the hunting mechanism is used. The wolves first encircle the pray. It is indicated mathematically using the following equations [26].

$$\begin{array}{cc} \vec{D} & =|\vec{C}.{\vec{X}}_{prey(t)}-{\vec{X}}_{wolf(t)}|\\ {\vec{X}}_{wolf(t+1)} & ={\vec{X}}_{prey(t)}-\vec{A}.\vec{D} \end{array}$$

*X*_*prey*_ is the position vector of the prey. *X*_*wolf*(*t*)_ is the current position of a particular wolf.*X*_*wolf*(*t*+1)_ is the position vector in the next iteration of the particular wolf.$\vec{A}$,$\vec{C}$ are coefficients which are calculated like following.

$$\begin{array}{cc} \vec{A} & =2.\vec{a}.{\vec{r}}_{1}-\vec{a}\\ \vec{C} & =2.{\vec{r}}_{2} \end{array}$$

${\vec{r}}_{1}$, ${\vec{r}}_{2}$ are position vectors which vary from [0, 1] while value of $\vec{a}$ decrease from 2,0. The positions of *α*, *β*, *δ* and *ω* are calculated according to the following equations.

$$\begin{array}{cc} {\vec{D}}_{\alpha } & =|{\vec{C}}_{1}.{\vec{X}}_{\alpha }-\vec{X}|\\ {\vec{D}}_{\beta } & =|{\vec{C}}_{2}.{\vec{X}}_{\beta }-\vec{X}|\\ {\vec{D}}_{\gamma } & =|{\vec{C}}_{3}.{\vec{X}}_{\gamma }-\vec{X}|\\ X_{1} & ={\vec{X}}_{\alpha }-{\vec{A}}_{1}.{\vec{D}}_{\alpha }\\ X_{2} & ={\vec{X}}_{\beta }-{\vec{A}}_{2}.{\vec{D}}_{\beta } \end{array}$$

Using these equations, the optimization algorithm is processed [27] The algorithm used in GWO using these equations can be shown in a pseudo code as in algorithm 1

**Algorithm 1** Pseudo code for the optimization of hyperparameters using GWO

**Input:** Model for x, x = (*x*_1_, *x*_2_, ……, *x_d_*) set of hyperparameters

**Output:** optimal set of hyperparameters

 *Initialization*:

1: Generate an initial population of grey wolves *x_i_*: *i* = (1, 2, ….., *n*)

2: Initialize a,A and C

3: Initialize social hierarchy

  *x*_*α*_ = best search agent

  *x*_*β*_ = second best search agent

  *x*_*γ*_ = third best search agent

4: **while** *r* <= *iterations* **do**

5:  **for** *i* = 1 to *n* **do**

6:   update position of *x*_*i*_

7:  **end for**

8:  update a, A and C

9:  calculate fitness of all search agents

10:  update *x*_*α*_, *x*_*β*_, *x*_*γ*_

11:  *r* = *r* + 1

12: **end while**

13: **return** *x*_*α*_

As shown in the algorithm 1, after creating the population, or the pack of wolves(X), the alpha beta and omega search agents are defined. After initializing A, C, a; for a given number of iterations the suitable search agents are derived. The criteria which is used to measure the performance of the function and using that criteria, the given GWO algorithm will function. The following were considered as the hyper parameters of the LSTM neural network [28]. These values for the parameters were considered as the most suitable for the analysis with the prior experiments conducted. A sample space is created using the values of each hyper parameters from the sample space of number of epoch (200, 500, 1000), batch size (100, 200, 500), activation functions sigmoid, tanh, relu, linear), optimizer (adam, rms). Then that sample is mapped into x = 0, 1, ……… n values which would be used as the population in the GWO algorithm. Each x value represents a combination of values of each hyper parameters. In the algorithm, according to the given x value, 0:n the value of the hyper parameters is fed into the neural network created. As the benchmark or the performance measuring mechanism, the mean squared error of the prediction and actual values is considered [29]. The process is conducted in a manner it would reduce the mean squared error of the function. At the end of the function, the best fit solution—alpha is given and it is used as the set of values used as the hyper parameters.

## Results

Data from only 19 districts were available for analysis asrecords of Kalutara, Matale, Matara, Kilinochchi, Mulaitvu andKegalle districts were not available for analysis.

**Fig 3:**
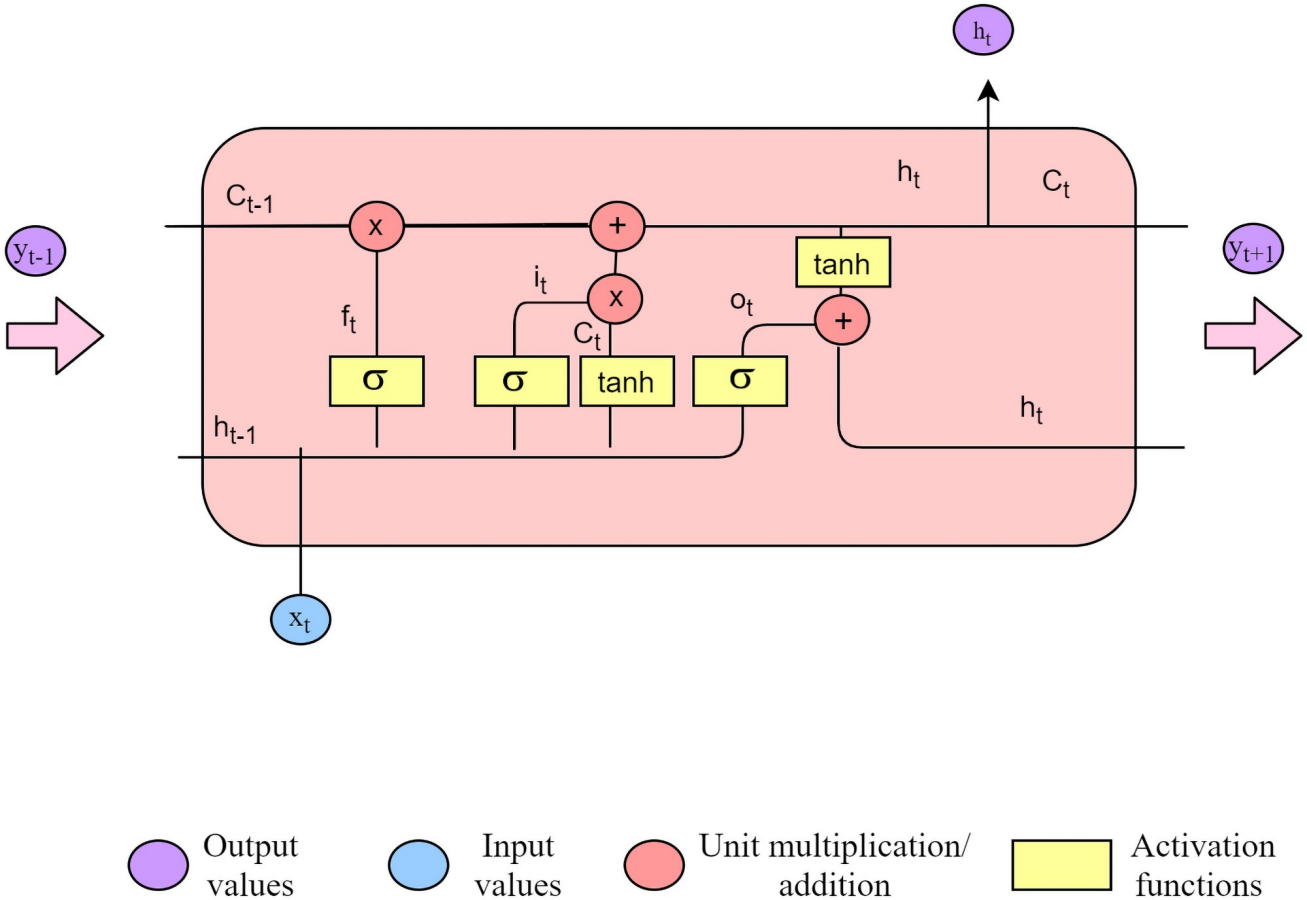
Variation of population density, rainfall, temperature and wind speed in the considered time period (2010-2019).

The Fig 3 show the variation population density and the average values of rainfall, temperature and wind speed of each month between 2010-2019 of Sri Lanka. The fluctuation of the population density over different months is negligible as shown in the Fig 3. Table 1 demonstrates the number of cases of dengue reported to each district during the study period.

**Table 1:** The number of dengue cases reported during the considered period of time in each district. The table is arranged from descending order from highest number of reported cases to the lowest.

District	Number of cases
Colombo	122751
Gampaha	77799
Kandy	33523
Ratnapura	30200
Kurunagala	28784
Jaffna	21572
Batticaloa	17721
Galle	17027
Puttalam	16906
Badulla	10165
Trincomalee	9453
Hambantota	8687
Anuradhapura	8035
Monaragala	7050
Polonnaruwa	4524
Vavuniya	3142
NuwaraEliya	3107
Ampara	2429
Mannar	2387

Data from Ampara district is presented to highlight the accuracy of the model. Similar results were seen in the remaining 18 districts. There were two main findings in this study.

**Fig 4:**
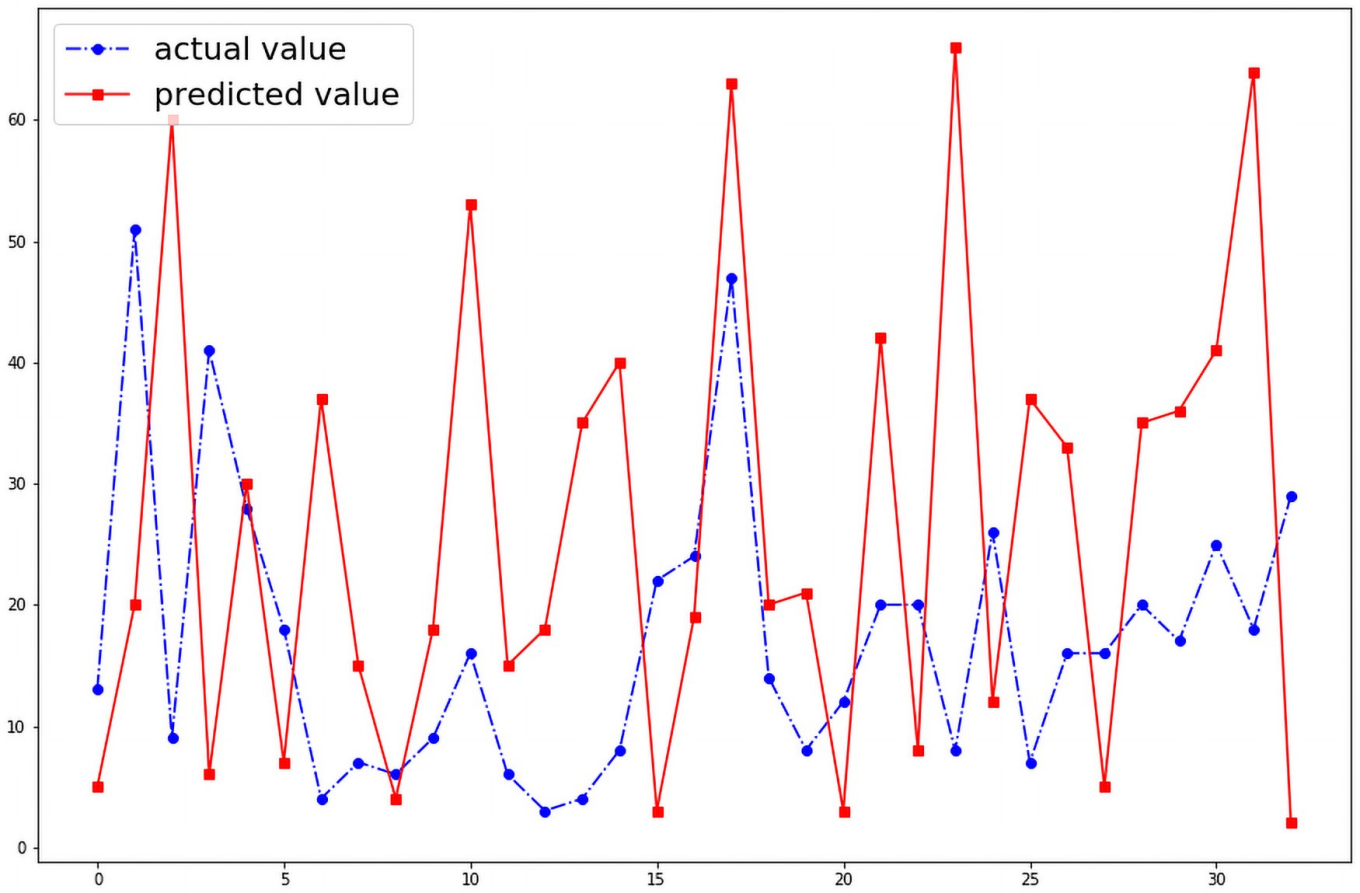
Without optimization (RMSE: 25.45).

**Fig 5:**
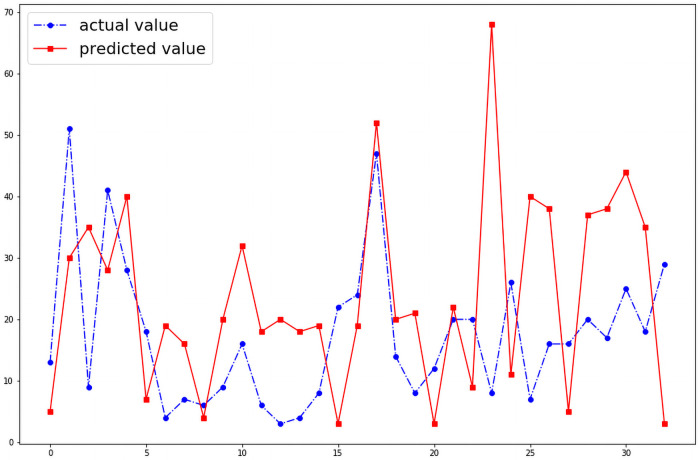
With optimization (RMSE: 20.45).

**Fig 6:**
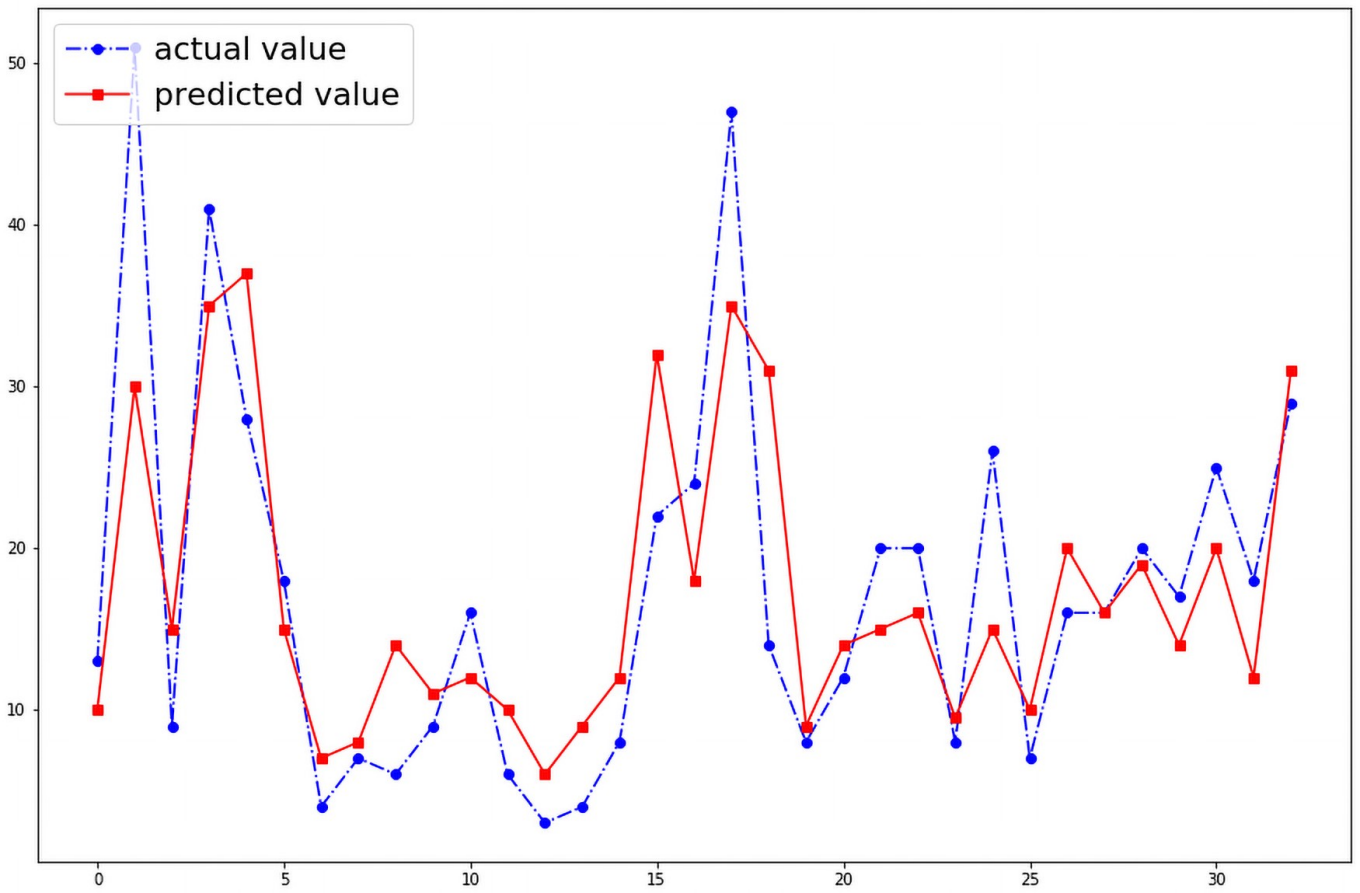
With population (RMSE: 18.32).

**Fig 7:**
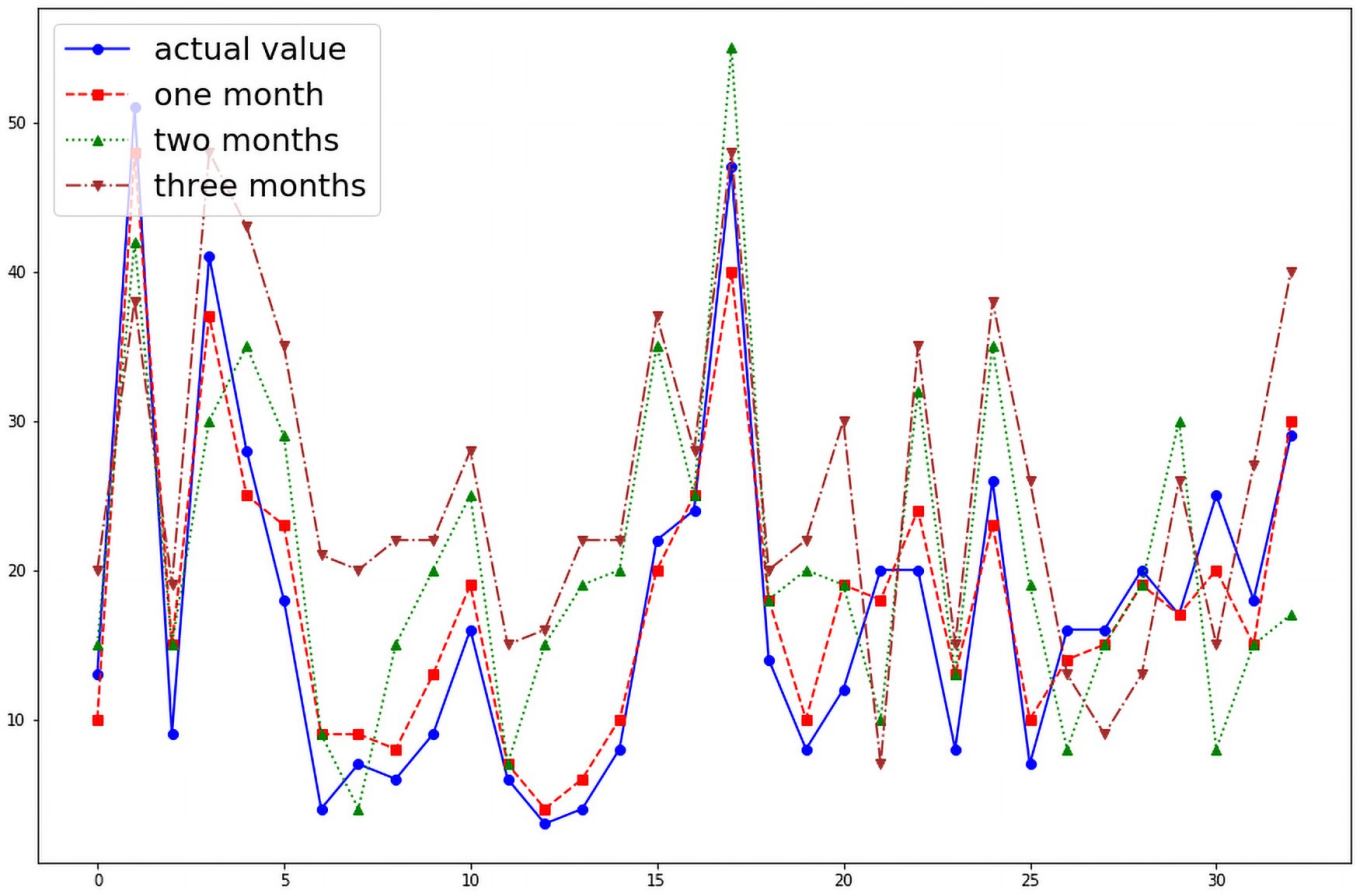
Comparison of model’s ability to predict the number of new cases in the succeeding months.

1. We were able to predict the number of cases of dengue fever in any given month of the 19 districts. Without optimizing the hyper parameters the model shows a wide variation between the actual and the predicted number of cases in a given month as shown in the Fig 4. However, when the model’s hyper parameters were optimized with GWO algorithm, the performance of the model improved as shown in the Fig 5. When the model was reanalyzed using population density as an input variable as a feature, the performance of the model further improved significantly which is illustrated by the Fig 6.
2. The model is able to predict the number of cases of dengue fever in the succeeding months. Further analysis was conducted to test the model’s ability to predict new cases in the succeeding months. The model demonstrated that it best predicts new cases in the immediate month after the index month. The Fig 7 shows the comparison of predicted and actual values of the model.

**Fig 8:**
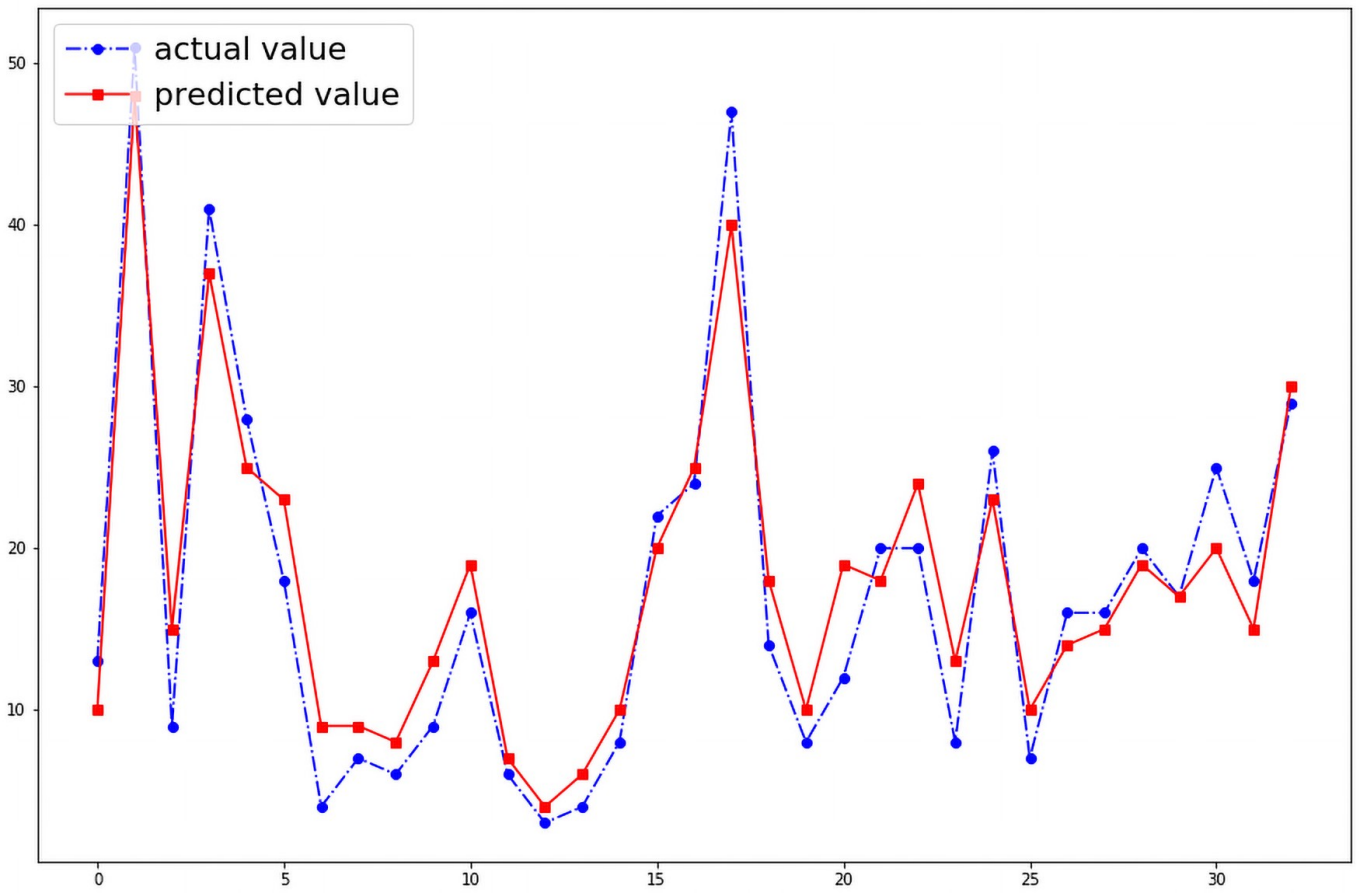
Final model (RMSE: 10.84).

The final model incorporating all input variables is depicted in Fig 8. This model shows that when all input variables are incorporated the model performs with significant accuracy in all the districts. Table 2 shows the RMSE (Root Mean Squared Value) values for each district.

**Table 2:** The RMSE values of each districts.

District	RMSE value
Ampara	10.84
Anuradhapura	21.25
Badulla	31.32
Batticaloa	65.43
Colombo	36.45
Galle	26.65
Gampaha	52.64
Hambantota	22.79
Jaffna	39.62
Kandy	32.74
Kurunagala	36.51
Mannar	13.45
Monaragala	12.64
NuwaraEliya	10.13
Polonnaruwa	11.62
Puttalam	26.89
Ratnapura	36.27
Trincomalee	27.33
Vavuniya	10.14

## Discussion

The developed model demonstrates that using the existing data of a given month, the number of cases can be estimated with minimal error. The results obtained from the model proves our hypothesis that a mathematical relationship exists between number of cases of dengue fever and environmental factors such as rain, humidity and wind speed. This relationship is very strong in that the predicted number of cases and actual number of cases have a very narrow RMSE. As the study was conducted per district wise, the effect of intra district variances does not affect the model created. The variation of socioeconomic factors have previously not been associated with number of cases and hence is unlikely to significantly affect the model. Our results also indicated that using data from a given month, the potential number of new cases expected in the immediate next month too could be predicted. The RMSE for this prediction can be as narrow as 10.14 in districts with low incidence of dengue fever. Further, the prediction for the second and third succeeding months too can be predicted. This is the first study that looked at developing a mathematical model to predict dengue fever in a tropical country. Previously, Dharmawardana. K et al [30] demonstrated that there may be a relationship between dengue and population mobility. However, this study used data on use of mobile phones as their method to calculate to extrapolate the population. A major drawback of this study is that many people use more than one telephone and their assumptions therefore may be subject to errors. Further, their study was conducted over a period of one year. In contrast, our study was conducted using 19 of the 26 districts over period of 10 years and used actual population data. This study shows that epidemics can be predicted using existing data. This study could be used by the epidemiology units of to countries such as Sri Lanka to predict epidemics and allocate appropriate resources to areas of high incidence and also take appropriate preventive measures through health education and other methods. This research is based on the temporal data of Sri Lanka from 2010—2019. Hence to predict time series data models such as SVR, MLP can be used. But the highest performance is shown by the LSTM neural network. Many research studies have been conducted to study the usage of optimization algorithms to tune the hyper parameters of a machine learning model. Through this research, the mechanism where the GWO algorithm could be used to optimize the hyperparameters of a LSTM neural network is introduced. As shown in the Figs 4 and 5 usage of the GWO algorithm to optimize the network, increased the performance of the model.

There were missing weather data entries in the data obtained from meteorological department of Sri Lanka. However, that limitation was overcome by preprocessing data before building the machine learning model. Although the weather data, population data as well as the number of dengue cases are collected properly by the relevant sources, there is always a slight chance of misinterpretation of data.
